# Tolerances For Layer Thicknesses in Dielectric Multilayer Coatings and Interference Filters

**DOI:** 10.6028/jres.064A.047

**Published:** 1960-12-01

**Authors:** Klaus D. Mielenz

## Abstract

A theory is developed for dielectric multilayer coatings in which the layers depart from calculated thickness. The theory is applied to alternating systems of quarter wave layers of ZnS and MgF_2_. The effects of thickness errors are: (1) A shift of the wavelength at which maximum reflectance occurs; and (2) a change in phase shift upon reflection. The magnitude of these effects, and also their dependence on various parameters, are determined. Statistical tolerances for layer thicknesses are computed for given tolerances on the multilayer performance. The accuracy required for producing dielectric interference filters is up to about 40 times higher than the accuracy sufficient for the production of dielectric mirrors and beam splitters. Various techniques of experimentally controlling film thicknesses, and their accuracies, are discussed. The production of mirrors and beam splitters deviating from theoretical maximum reflectance by only 1 percent seems to be possible with Dufour’s simple single photocell method of monitoring film thicknesses. With more precise methods, such as those developed by Giacomo and Jacquinot, or Traub, the production of interference filters appears to be possible to within plus or minus one half their half widths.

## 1. Introduction

In the production of dielectric multilayer coatings, such as mirrors, beam splitters, or interference filters, it is important to control the thickness of the layers with an accuracy sufficient to attain experimentally the high performance of which these coatings are capable.

Heavens [1] 1 has calculated, in some few examples, the effect of errors in layer thicknesses on the energy reflected from high-reflecting multilayer coatings, and Giacomo [2] has done similar work on the effect of these errors on the phase change upon reflection from such coatings. Neither author, however, has derived the tolerances on individual layer thicknesses that may be allowed if the coating is to meet a given performance within certain explicitly specified limits.

Such tolerances are computed in this paper, and various techniques of monitoring layer thicknesses are compared from the point of view of these tolerances.

## 2. Basic Formulas

### 2.1 General Case

The electromagnetic field at the plane of incidence on a stack of *N* dielectric layers is, according to Koehler [3],

$$\left(\begin{array}{l} E\\ H \end{array}\right)=U_{1}U_{2}\ldots U_{N}\left(\begin{array}{l} 1\\ n_{s} \end{array}\right)$$ (1)

(*n_s_*=substrate index), with

$$U_{v}=\;\cos\;\beta_{v}\;U+i\;\sin\;\beta_{v}{ℜ}_{v},$$ (2)

$$U=\left(\begin{array}{ll} 1 & 0\\ 0 & 1 \end{array}\right),\;{ℜ}_{v}=\left(\begin{array}{ll} 0 & 1/n_{v}\\ n_{v} & 0 \end{array}\right).$$ (3)

Here,

$$\beta_{v}=(2\pi /\lambda )n_{v}d_{v},\;v=1,2,\ldots N$$ (4)

(*n_v_* = refractive index, *d_v_* = geometrical thickness, *λ*= vacuum wavelength) represents the optical thickness of the *v*th layer.

The amplitudes transmitted and reflected by the multilayer are

$$T=(2\sqrt{n_{0}n_{s}})/C^{+}=\sqrt{\tau }\;e^{i\theta },$$ (5)

$$R=C^{-}/C^{+}=\sqrt{\rho }\;e^{i\Phi },$$ (6)

where *τ* and *ρ* denote energy transmittance and reflectance, and with

$$C^{\pm }=n_{0}E\pm H$$ (7)

(*n*_0_ = index of medium of incidence). Hence

$$\tau =\frac{4n_{0}n_{s}}{{\left(ℛC^{+}\right)}^{2}+{\left(IC^{+}\right)}^{2}}$$ (8)

and, for truly dielectric layers,

ρ=1−τ. (9)

Furthermore,

$$\Phi =\theta +\alpha ,\;\tan\;\theta =-IC^{+}/ℛC^{+},\;\tan\;\alpha =IC^{-}/ℛC^{-}.$$ (10)

Here, ℛ and ***I*** denote real and imaginary part, respectively.

Of the two phase angles *θ* and Φ, only Φ, the phase change upon reflection, is of practical significance in most cases, as, for instance, for the energy transmitted by a Fabry-Perot interferometer (see eq (60)). Throughout this paper, therefore, *θ* is an auxiliary quantity only that is needed for the computation of Φ.

### 2.2. Alternating Multilayers

Consider an odd number, *N* =2*m*+1, of layers of nominally equal optical thickness, alternately of high index *n_H_* and low index *n_L_*, with a high index on the outside. If all layers are a quarter wave thick at a wavelength *λ*_0_,

$$\beta_{1}=\beta_{2}=\cdots =\beta =(\pi /2)(\lambda_{0}/\lambda ),$$ (11)

eq (2) may be written as

$$\left(\begin{array}{l} E\\ H \end{array}\right)=[S_{m}(x)U_{H}-S_{m-1}(x)U_{L}^{-1}]\left(\begin{array}{l} 1\\ n_{s} \end{array}\right),$$ (12)

with

$$U_{H}=\;\cos\;\beta \;U+i\;\sin\;\beta \;{ℜ}_{H},$$ (13)

$$U_{L}^{-1}=\;\cos\;\beta \;U-i\;\sin\;\beta \;{ℜ}_{L}.$$ (14)

*S_m_* and *S_m_*_−1_ are Chebychev polynomials of the argument

$$x=2-\frac{{\left(n_{H}+n_{L}\right)}^{2}}{n_{H}n_{L}}{\;\sin\;}^{2}\beta ,$$ (15)

defined by

$$\begin{array}{ll} S_{m-1}(x)=\;\sin\;m\gamma /\;\sin\;\gamma , & x=2\;\cos\;\gamma , \end{array}$$ (16)

etc., see [4]. From eqs (12) to (14), and (7),

$$C^{\pm }=A^{\pm }\;\cos\;\beta +iB^{\pm }\;\sin\;\beta ,$$ (17)

with

$$\begin{array}{ll} A^{\pm }=K^{\pm }(S_{m}-S_{m-1}), & K^{\pm }=n_{0}\pm n_{s}, \end{array}$$ (18)

$$\begin{array}{ll} B^{\pm }=P^{\pm }S_{m}+Q^{\pm }S_{m-1}, & P^{\pm }=n_{0}n_{s}/n_{H}\pm n_{H},\\ & Q^{\pm }=n_{0}n_{s}/n_{L}\pm n_{L}. \end{array}\}$$ (19)

Hence, eqs (8), (9), (10), and (17) yield

$$\tau =1-\rho =\frac{4n_{0}n_{s}}{{\left(A^{+}\;\cos\;\beta \right)}^{2}+{\left(B^{+}\;\sin\;\beta \right)}^{2}}$$ (20)

$$\begin{array}{l} \;\tan\;\theta =-(B^{+}/A^{+})\;\tan\;\beta ,\\ \;\tan\;\alpha =(B^{-}/A^{-})\;\tan\;\beta ,\\ \;\Phi =\theta +\alpha . \end{array}\}$$ (21)

## 3. Multilayers With Layer Thickness Errors

### 3.1. General Case

If, in the multilayer, the optical thicknesses *n_v_d_v_* of the films differ from their calculated values by slight amounts Δ*_v_* = Δ(*n_v_d_v_*), the (*β_v_*)′s of section 2.1 must be replaced by

$$\beta_{v}^{\prime }=\beta_{v}+\Delta \beta_{v},\;\text{with}\;\Delta \beta_{v}=(2\pi /\lambda )\Delta_{v}.$$ (22)

Let Δ*β_v_* be sufficiently small, so that

$$\;\cos\;\beta_{v}^{\prime }=\;\cos\;\beta_{v}-\Delta \beta_{v}\;\sin\;\beta_{v},$$ (23)

$$\;\sin\;\beta_{v}^{\prime }=\;\sin\;\beta_{v}+\Delta \beta_{v}\;\cos\;\beta_{v}.$$ (24)

Then, the matrices $U_{v}$ will be changed to

$$U_{v}^{\prime }=U_{v}+\Delta \beta_{v}B_{v},$$ (25)

with $U_{v}$ from eq (3), and

$$B_{v}=-\;\sin\;\beta_{v}U+i\;\cos\;\beta_{v}\;{ℜ}_{v}.$$ (26)

Therefore, eq (2) is transformed into

$$\left(\begin{array}{l} E^{\prime }\\ H^{\prime } \end{array}\right)=U_{1}^{\prime }U_{2}^{\prime }\ldots U_{N}^{\prime }\left(\begin{array}{l} 1\\ n_{s} \end{array}\right)=\left(\begin{array}{l} E\\ H \end{array}\right)+\sum_{v=1}^{\lambda }\left(\begin{array}{l} \Delta E_{v}\\ \Delta H_{v} \end{array}\right),$$ (27)

with $\left(\begin{array}{l} E\\ H \end{array}\right)$ from eq. (2), and

$$\begin{array}{l} \left(\begin{array}{l} \Delta E_{v}\\ \Delta H_{v} \end{array}\right)=\Delta \beta_{v}D_{v}\left(\begin{array}{l} 1\\ n_{s} \end{array}\right),\\ \;D_{v}=U_{1}U_{2}\ldots U_{v-1}B_{v}U_{v+1}\ldots U_{N}. \end{array}\}$$ (28)

In this first-order approximation, accordingly, each Δ*β_v_* causes separate additive terms in *E* and *H*, so that

$$\begin{array}{l} {\left(C^{\pm }\right)}^{\prime }=n_{0}E^{\prime }\pm H^{\prime }\\ \;=(n_{0}E\pm H)+\sum_{v=1}^{N}\left(n_{0}\Delta E_{v}\pm \Delta H_{v}\right)\\ \;=C^{\pm }+\sum_{v=1}^{N}\Delta C_{v}^{\pm }. \end{array}\}$$ (29)

Thus, the individual errors Δ*β_v_* may be considered separately.

### 3.2. Alternating Multilayers

#### a. Incorrect High-Index Layer

Consider an error in thickness in one of the high-index layers, *v = 2k*+1. Then, eq (28) yields

$$D_{2k+1}={\left(U_{H}U_{L}\right)}^{k}B_{H}{\left(U_{L}U_{H}\right)}^{m-k}.$$ (30)

According to [4],

$${\left(U_{H}U_{L}\right)}^{n}=S_{n-1}U_{H}U_{L}-S_{n-2}U,$$

and therefore,

$$\begin{array}{l} D_{2k+1}=S_{k-1}S_{m-k-1}U_{H}U_{L}B_{H}U_{L}U_{H}\\ \;-S_{k-2}S_{m-k-1}B_{H}U_{L}U_{H}\\ \;-S_{k-1}S_{m-k-2}U_{H}U_{L}B_{H}\\ \;+S_{k-2}S_{m-k-2}B_{H}. \end{array}\}$$ (31)

From (13), (14), (15), and (26), the following identities can be derived:

$$\begin{array}{c} U_{H}U_{L}B_{H}U_{L}U_{H}=-[(x^{2}-x+1)U+(x-1)\\ ({ℜ}_{H}{ℜ}_{L}+{ℜ}_{L}{ℜ}_{H}]\;\sin\;\beta +i[{\left(x-1\right)}^{2}{ℜ}_{H}+(x-2){ℜ}_{L}]\;\cos\;\beta ,\\ B_{H}U_{L}U_{H}=-(xU+{ℜ}_{H}{ℜ}_{L})\;\sin\;\beta +i\left(x-1\right){ℜ}_{H}\;\cos\;\beta ,\\ U_{H}U_{L}B_{H}=-(xU+{ℜ}_{L}{ℜ}_{H})\;\sin\;\beta +i\left(x-1\right){ℜ}_{H}\;\cos\;\beta , \end{array}$$

with

$${ℜ}_{H}{ℜ}_{L}=\left(\begin{array}{cc} n_{L}/n_{H} & 0\\ 0 & n_{H}/n_{L} \end{array}\right),$$ (32)

$${ℜ}_{L}{ℜ}_{H}=\left(\begin{array}{cc} n_{H}/n_{L} & 0\\ 0 & n_{L}/n_{H} \end{array}\right).$$ (33)

These together with the following recurrence relation between Chebychev polynomials:

$$S_{n}=xS_{n-1}-S_{n-2},$$

provide

$$\begin{array}{l} D_{2k-1}=-[(S_{k}S_{m-k}-[x-1]S_{k-1}S_{m-k-1})U\\ \;+S_{m-k-1}(S_{k}-S_{k-1}){ℜ}_{H}{ℜ}_{L}\\ \;+S_{k-1}(S_{m-k}-S_{m-k-1}){ℜ}_{L}{ℜ}_{H}]\;\sin\;\beta \\ \;+i[(S_{k}-S_{k-1})(S_{m-k}-S_{m-k-1}){ℜ}_{H}\\ \;+(x-2)S_{k-1}S_{m-k-1}{ℜ}_{L}]\;\cos\;\beta . \end{array}\}$$ (34)

Thus, it follows from (28), (29), and (34),

$$\Delta C_{2k+1}^{\pm }=\Delta \beta_{2k+1}(A_{2k+1}^{\pm }\;\sin\;\beta +iB_{2k+1}^{\pm }\;\cos\;\beta ),$$ (35)

with

$$\begin{array}{l} A_{2k+1}^{\pm }=-K^{\pm }[S_{k}S_{m-k}-(x-1)S_{k-1}S_{m-k-1}]\\ \;-L^{\pm }S_{m-k-1}(S_{k}-S_{k-1})\\ \;-M^{\pm }S_{k-1}(S_{m-k}-S_{m-k-1}), \end{array}\}$$ (36)

$$\begin{array}{l} B_{2k+1}^{\pm }=P^{\pm }(S_{k}-S_{k-1})(S_{m-k}-S_{m-k-1})\\ \;+Q^{\pm }(x-2)S_{k-1}S_{m-k-1}, \end{array}\}$$ (37)

*K*^±^, *P*^±^, and *Q*^±^, from eqs (18) and (19), and

$$\begin{array}{l} \;L^{\pm }=n_{0}n_{L}/n_{H}\pm n_{s}n_{H}/n_{L},\\ M^{\pm }=n_{0}n_{H}/n_{L}\pm n_{s}n_{L}/n_{H}. \end{array}\}$$ (38)

#### b. Incorrect Low-Index Layer

Next, consider a thickness error in one of the low-index layers, *v = 2k*, so that

$$D_{2k}={\left(U_{H}U_{L}\right)}^{k-1}U_{H}B_{L}{\left(U_{H}U_{L}\right)}^{m-k}U_{H}.$$ (39)

Because of [4]

$${\left(U_{H}U_{L}\right)}^{n}U_{H}=S_{n}U_{H}-S_{n-1}U_{L}^{-1},$$

this is equal to

$$\begin{array}{l} D_{2k}=S_{k-1}S_{m-k}U_{H}B_{L}U_{H}-S_{k-2}S_{m-k}U_{L}^{-1}B_{L}U_{H}\\ \;-S_{k-1}S_{m-k-1}U_{H}B_{L}U_{L}^{-1}\\ \;+S_{k-2}S_{m-k-1}U_{L}^{-1}B_{L}U_{L}^{-1}. \end{array}\}$$ (40)

From (13), (14), (15), and (26), it can be shown that

$$\begin{array}{l} \;U_{H}B_{L}U_{H}=-[(x-1)U+{ℜ}_{H}{ℜ}_{L}+{ℜ}_{L}{ℜ}_{H}]\;\sin\;\beta \\ \;+i[(x-2){ℜ}_{H}+{ℜ}_{L}]\;\cos\;\beta ,\\ \;U_{L}^{-1}B_{L}U_{H}=-{ℜ}_{L}{ℜ}_{H}\;\sin\;\beta +i{ℜ}_{L}\;\cos\;\beta ,\\ \;U_{H}B_{L}U_{L}^{-1}=-{ℜ}_{H}{ℜ}_{L}\;\sin\;\beta +i{ℜ}_{L}\;\cos\;\beta ,\\ U_{L}^{-1}B_{L}U_{L}^{-1}=\;\sin\;\beta \;U+i{ℜ}_{L}\;\cos\;\beta , \end{array}$$

so that

$$\begin{array}{l} D_{2k}=-[([x-1]S_{k-1}S_{m-k}-S_{k-2}S_{m-k-1})U\\ \;+S_{k-1}(S_{m-k}-S_{m-k-1}){ℜ}_{H}{ℜ}_{L}\\ \;+S_{m-k}(S_{k-1}-S_{k-2}){ℜ}_{L}{ℜ}_{H}]\;\sin\;\beta \\ \;+i[(x-2)S_{k-1}S_{m-k}{ℜ}_{H}\\ \;+(S_{k-1}-S_{k-2})(S_{m-k}-S_{m-k-1}){ℜ}_{L}]\;\cos\;\beta . \end{array}\}$$ (41)

Eventually, (28), (29), and (41) provide the result

$$\Delta C_{2k}^{\pm }=\Delta \beta_{2k}(A_{2k}^{\pm }\;\sin\;\beta +iB_{2k}^{\pm }\;\cos\;\beta ),$$ (42)

with

$$\begin{array}{l} A_{2k}^{\pm }=-K^{\pm }[(x-1)S_{k-1}S_{m-k}-S_{k-2}S_{m-k-1}]\\ \;-L^{\pm }S_{k-1}(S_{m-k}-S_{m-k-1})\\ \;-M^{\pm }S_{m-k}(S_{k-1}-S_{k-2}), \end{array}\}$$ (43)

$$\begin{array}{l} B_{2k}^{\pm }=P^{\pm }(x-2)S_{k-1}S_{m-k}\\ \;+Q^{\pm }(S_{k-1}-S_{k-2})(S_{m-k}-S_{m-k-1}), \end{array}\}$$ (44)

and coefficients *K*^±^, *L*^±^, etc., already known.

#### c. Validity of Approximation

**Figure 1:**
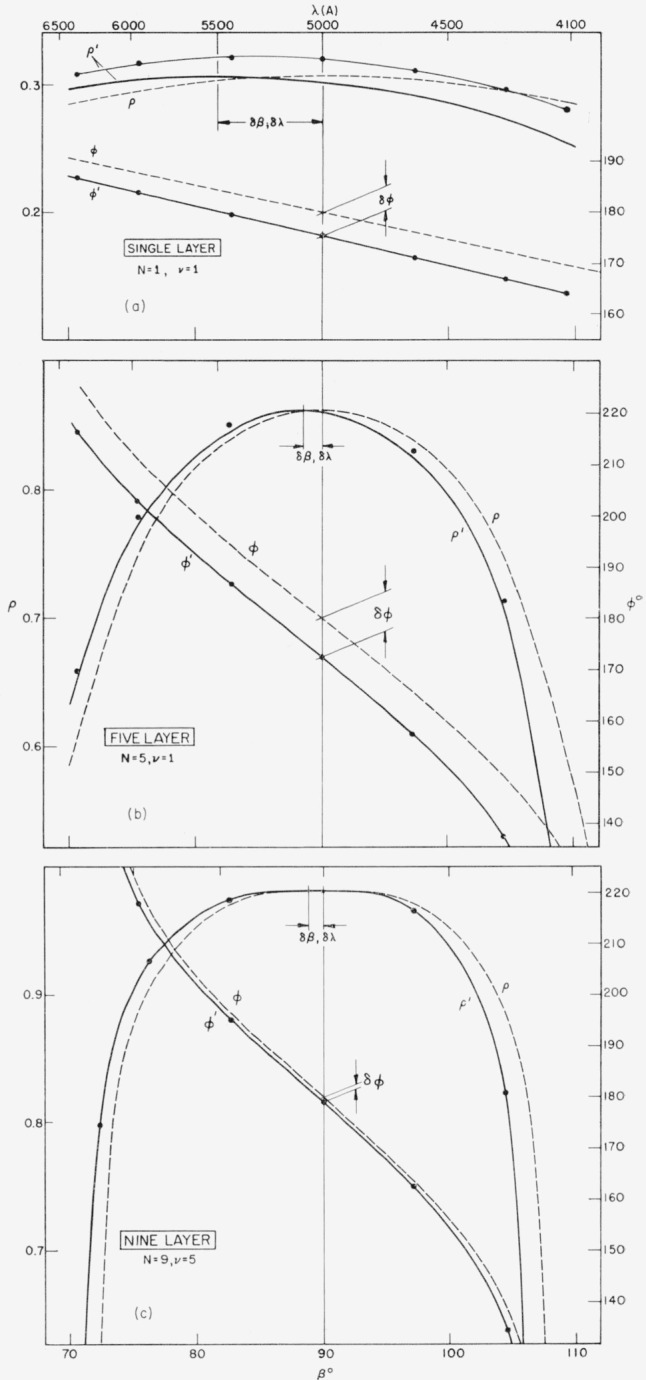
Effects of errors in layer thickness on reflectance ρ and phase shift upon reflection Φ of alternating quarter wave layers of *ZnS* and *MgF*_2_ between air and a glass substrate (*n*_0_=1, *n_H_* = 2.3, *n_L_* = 1.38, *n_s_* = 1.52), *ZnS* bottom layer (a) Single film of ZnS, off by 10 percent, (b) five-layer with top layer (*v* = 1) off by 10 percent, and (c) nine-layer with central layer (*v* = 5) off by 10 percent. Solid lines: exact values. Dots: approximate values. Wavelength scale for λ_0_= 5000 A. For comparison broken lines show ρ and Φ for the respective correct multilayers. Effects of errors are wavelength shift δ*β*, or δλ, of maximum reflectance, and change δΦ in phase shift upon reflection.

Introducing into (29) and (6) the Δ*C*’s of eqs (35) and (42), one may calculate the desired amplitudes,

$$R^{\prime }=\sqrt{\rho^{\prime }}\;e^{i\Phi^{\prime }},$$ (45)

reflected from multilayers in which one or several, film thicknesses differ from their calculated values *β* by given amounts Δ*β*. The mathematics developed is based upon the assumption of small Δ*β*’s, made by eqs (23) and (24). In order to establish the validity of this approximation, the *ρ*′ and Φ′-values of three different zinc sulphide-magnesium fluoride multilayers with in each case one film deviating by Δ*β* = 10 percent were calculated both from the formulas derived here and also by exact computation. A comparison of results is given in figures 1a, b, and c. Agreement of exact and approximate *ρ*′-values, while poor for the single film (where an approximation is hardly needed), is good for higher numbers of layers (within 0.005 for the 5-layer, 0.001 for the 9-layer). The Φ′-values are in almost perfect agreement (within 0.1°) in all cases, including the monolayer.

## 4. Effects of Errors in Layer Thicknesses

Besides showing *ρ*′ and Φ′ for various nonideal coatings, figures 1a, b, and c also show *ρ* and Φ for the respective ideal coatings, thus making apparent the results of thickness errors:

Contrary to what might be expected, such errors do not result in a noticeable decrease in reflectance at the central wavelength *λ*_0_, see [1]. This is illustrated once more by table 1; in the examples chosen, a 10 percent error causes a decrease ranging from only 0.004 for a single film to 0.001 for a 9-layer.^2^

The noticeable results of incorrect layer thicknesses are a parallel shift of the *ρ* and Φ-curves.

Maximum reflectance $\rho_{0}^{\prime }$ occurs at *β* = 90° + *δβ_v_*, or at a wavelength *λ* = *λ*_0_+*δλ_v_*, instead of at *β* = 90°, or *λ* = *λ*_0_. All other *ρ*′s are shifted correspondingly. To find *δβ_v_*, plot *ρ*′ as a function of *β*, and read the displacement *δβ_v_* of the maximum. Then, eq (11) provides

$$\begin{array}{l} 90{}^{\circ}+\delta \beta_{v}=90{}^{\circ}\lambda_{0}/(\lambda_{0}+\delta \lambda_{v}),\\ \;\;\text{or}\;\delta \lambda_{v}=-\lambda_{0}\delta \beta_{v}/(90{}^{\circ}+\delta \beta_{v}). \end{array}\}$$ (46)

The phase angles Φ′ differ from their nominal values Φ by amounts *δ*Φ*_v_* that practically are constants over a wide wavelength range,

$$\Phi^{\prime }=\Phi +\delta \Phi_{v}.$$ (47)

Since the nominal values are Φ = 180° for *β* = 90°, *δ*Φ*_v_* is found by computing Φ′ at *β* = 90°, only, and taking the difference to 180°. This is readily done because of the simplified expressions of C^±^ and Δ*C*^±^ at that particular *β*.

Note that, for the multilayers with thickness errors the values of *β* at which maximum reflectance occurs are distinct from those at which the phase change upon reflection is 180°. The five layer of figure 1b, for instance, exhibits maximum reflectance at *β* = 88.5°, and 180° phase change at *β* = 86.0°, the two being as much as 2.5° or, for *λ*_0_ = 5000 A, 135 A apart. Therefore, Φ = 180° is no criterion for maximum reflectance.

**Figure 2:**
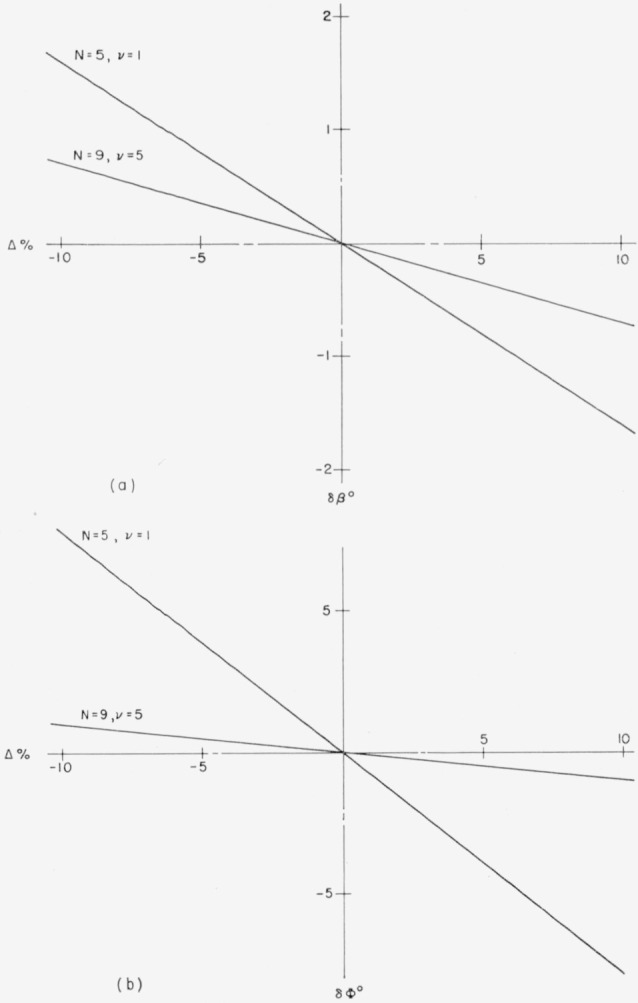
Dependence of (*a*) δ*β* and (*b*) δΦ upon δΔ. *N* = 5, *v* = 1:5-layer with wrong top layer; *N* = 9, *v* = 5: 9-layer with wrong central layer. *ZnS*-*MgF*_2_-films.

Figures 2 a and b show how, in two typical examples, *δβ_v_* and *δ*Φ*_v_* depend upon the magnitude of the thickness error Δ (*n_v_d_v_*). The relationship is a straight proportionality

$$\delta_{v}=a_{v}\Delta_{v},$$ (48)

where *δ_v_* stands for either *δβ_v_* or *δ*Φ*_v_*, *a_v_* for coefficients *a_v_*(*β*) and *a_v_*(Φ), and Δ*_v_* for Δ (*n_v_d_v_*).

As, in general, the thickness of more than one layer will be in error, it is important to know the total errors *δβ* and *δ*Φ produced by simultaneously occurring Δ*_v_*’s.

For three examples chosen at random, both the individual *δ_v_*’s as well as the total *δ*’s were computed, using eq (29) for the latter. These computations, of which the result is given in table 2, provide with good accuracy

$$\delta =\sum_{v=1}^{N}\delta_{v}$$ (49)

or, with (48),

$$\delta =\sum_{v=1}^{N}a_{v}\Delta_{v},$$ (50)

*δ* standing for either *δβ* or *δ*Φ. For *δ* = *δ*Φ, (48) is in accordance with Giacomo [2].

**Figure 3:**
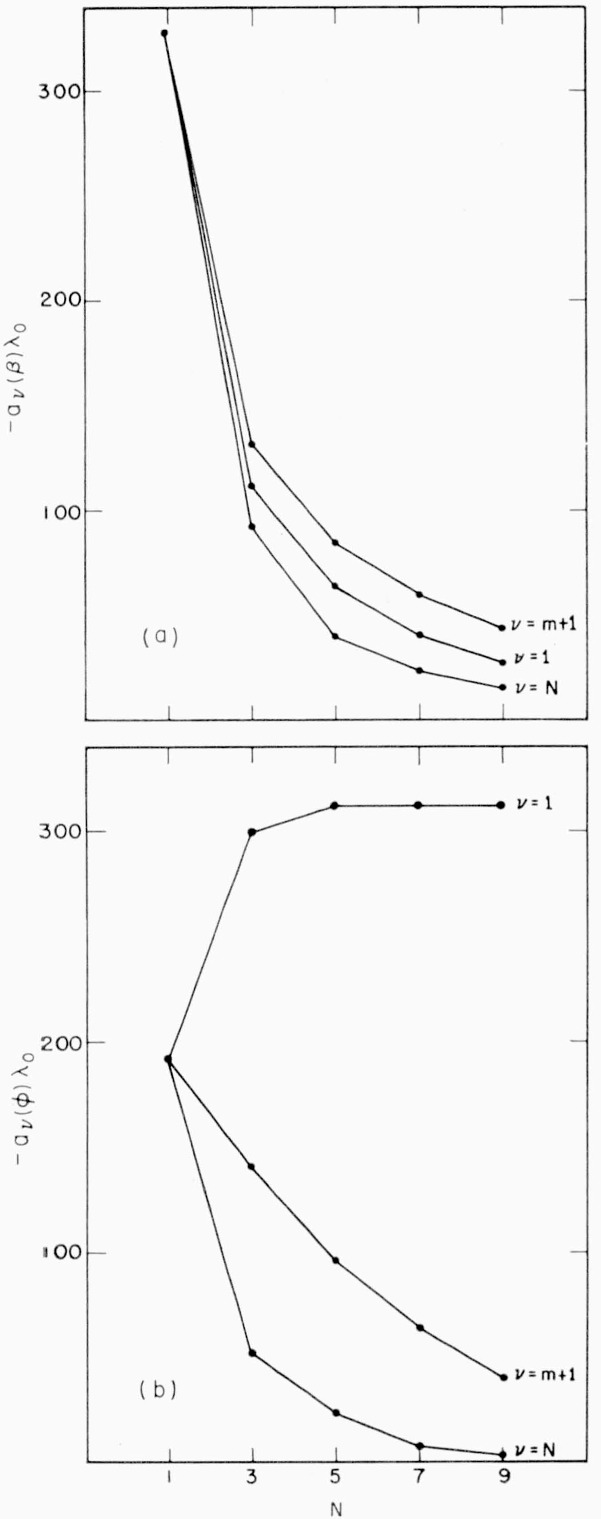
a, b. *a*_v_(β) and *a*_v_(Φ) as functions of *N* and v for *ZnS*-*MgF*_2_-multilayers.

For a number of stacks of alternating zinc sulphide and magnesium fluoride layers between air and a glass substrate with, in each case, an error of +10 percent in one of the layers, *δβ_v_* and *δ*Φ*_v_*, as well as *a_v_*(*β*) and *a_v_*(Φ), were computed. In figures 3 a and b, the *a_v_*’s are plotted versus the number, *N* = 2*m* + 1, of films in the stack. For either *a_v_*, the three curves show the effect of thickness errors occurring in the top, central, or bottom layer (*v* = 1, *m* + 1, or *N*, respectively). With the exception of *a*_1_(Φ), all *a_v_*’s tend to decrease as *N* increases.

**Figure 4:**
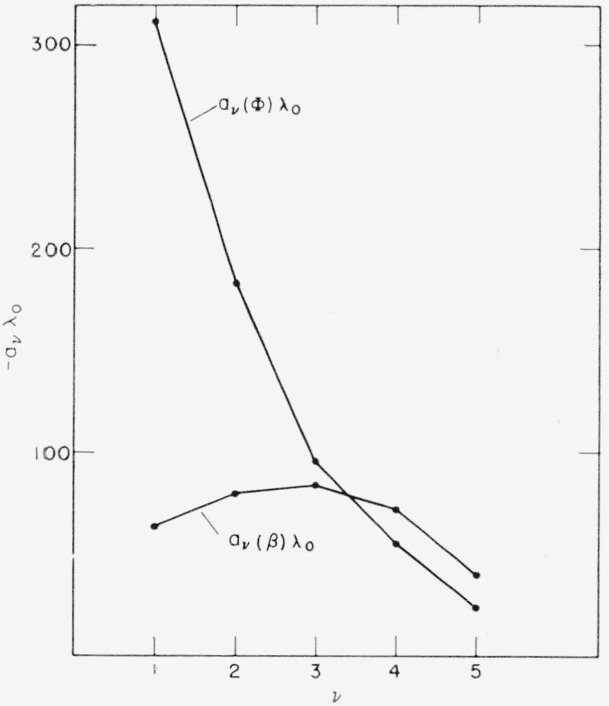
Dependence of *a*_v_(β) and *a*_v_(Φ) upon v for a *ZnS*-*MgF*_2_-5-layer.

According to figures 3a, b, and furthermore to figure 4, the *a_v_*’s also show a pronounced dependence upon *v*; i.e., upon where in the stack of layers the incorrect one is located:

The wavelength shift of the reflection maximum, or *a_v_*(*β*), is greatest if caused by the central layer, smallest for the bottom layer, and intermediate for the top layer. This result is not in agreement with Heavens’ statement [1] that the effect is greatest for the top layer, apparently because Heavens did not include in his treatment others than top or bottom layers.

The dependence of *a_v_*(Φ) upon *v* is different. Here, the effect increases steadily from the bottom towards the top layer, which is in agreement with Giacomo’s result [2].

## 5. Statistical Tolerances

### 5.1. Propagation of Errors

The observed general trend of *δβ_v_* and *δ*Φ*_v_* to decrease with increasing *N* does not imply that monitoring film thicknesses becomes easier as the number of films increases. It must also be taken into account that at high *N′*s the production of the multilayer requires control of a larger number of layers, so that there is an increased number of sources of error.

Let ± *s*(Δ*_v_*) be the random thickness error (standard deviation) of the *v*th. film, determined by sampling *Z* multilayers,

$$s^{2}(\Delta_{v})=\left(\sum_{i}\Delta_{vi}^{2}\right)/(Z-1)$$ (51)

(*i* = 1,2, … *Z*), and *± s* (*δ*) the standard deviation from the multilayer performance,

$$s^{2}(\delta )=\left(\sum_{i}\delta_{i}^{2}\right)/(Z-1),$$ (52)

with *δ* standing for either *δβ* or *δ*Φ. Equation (50) and the law of propagation of errors then provide

$$s^{2}(\delta )=\sum_{v=1}^{N}a_{v}^{2}s^{2}(\Delta_{v}).$$ (53)

Assume that monitoring the film thickness is equally difficult for each layer; i.e., that *s* (Δ*_v_*) is independent of *v*.

$$s(\Delta_{v})=s(\Delta ).$$ (54)

Hence,

$$s^{2}(\delta )=A^{2}s^{2}(\Delta ),$$

or

s(Δ)=s(δ)/A, (55)

with

$$A^{2}=\sum_{v=1}^{N}a_{v}^{2}.$$ (56)

By means of (55) it is now possible to determine within what limits ± *s* (Δ) each film thickness has to be controlled so that, on the basis of standard deviations, the finished multilayer will stay within a given tolerance ± *s* (*δ*).

**Figure 5:**
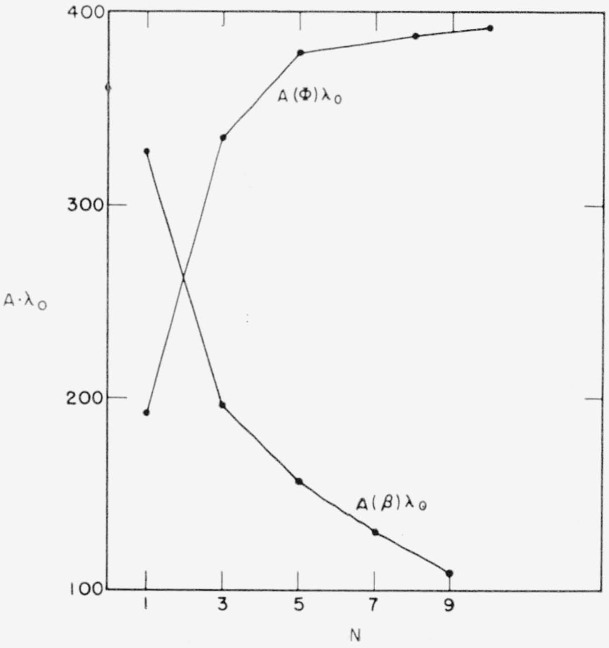
*A*(β) and *A*(Φ) as functions of *N* for *ZnS*-*MgF*_2_-multilayers.

For the accuracy required, *A*(*β*) and *A*(Φ), not a (*β_v_*) and a (Φ*_v_*), are the determining factors. *A*(*β*) and A (Φ) are plotted versus *N* in figure 5 showing that, besides being quite different in magnitude, *A*(*β*) is a rapidly decreasing and *A*(Φ) a rapidly increasing function of *N*.

These important differences between the effects of thickness errors upon the intensities of reflected waves and upon their phases lead to a separate consideration of the two cases; i.e., the “intensity” and the “phase” case.

### 5.2. Intensity Case: Mirrors and Beam Splitters

**Figure 6:**
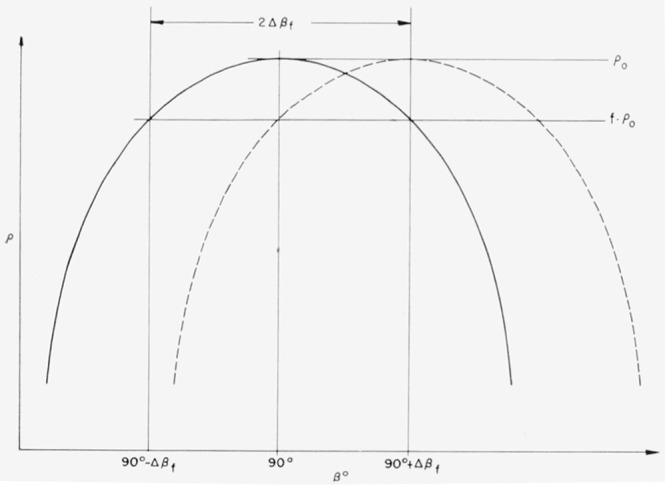
*f*-width 2Δβ*_f_* of multilayers and permissible wavelength shift of the reflection maximum.

Consider a multilayer designed to render a certain maximum reflectance *ρ*_0_ at a wavelength *λ*_0_. Let

$$\pm \Delta \rho =(1-f)\rho_{0}$$ (57)

be the permissible deviation from *ρ*_0_, with *f*<1. If, then, 2 *β_f_* is the “*f*—width” of the ideal multilayer; i.e., the width of the range of *β*′s for which *fρ*_0_≤ *ρ*≤*ρ*_0_; it follows immediately from figure 6 that the permissible wavelength shift of the reflection maximum is

$$\delta \beta =\pm \Delta \beta_{f}.$$ (58)

With this value substituted for *s* (*δβ*), eq (55) then provides, for the thickness tolerance,

$$s(\Delta )=\pm \Delta \beta_{f}/A(\beta ).$$ (59)

**Figure 7:**
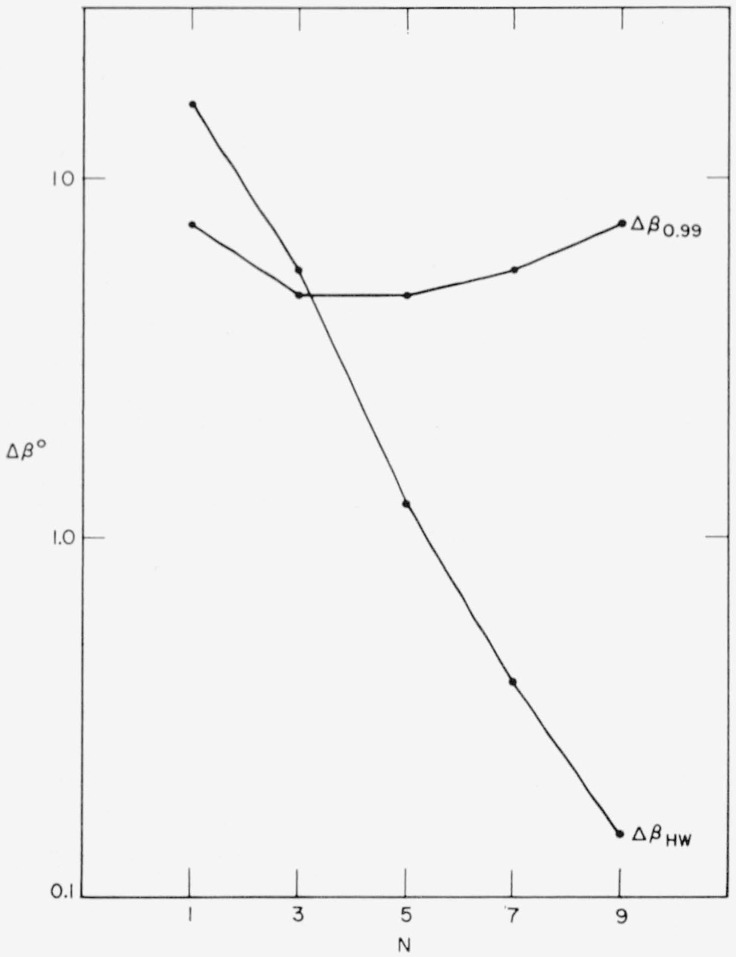
0.99-widths of *ZnS*-*MgF*_2_-multilayers versus number of layers *N*. Half widths of first-order interference filters having N alternating *ZnS*-*MgF*_2_-layers on each side of the (air) spacer.

**Figure 8:**
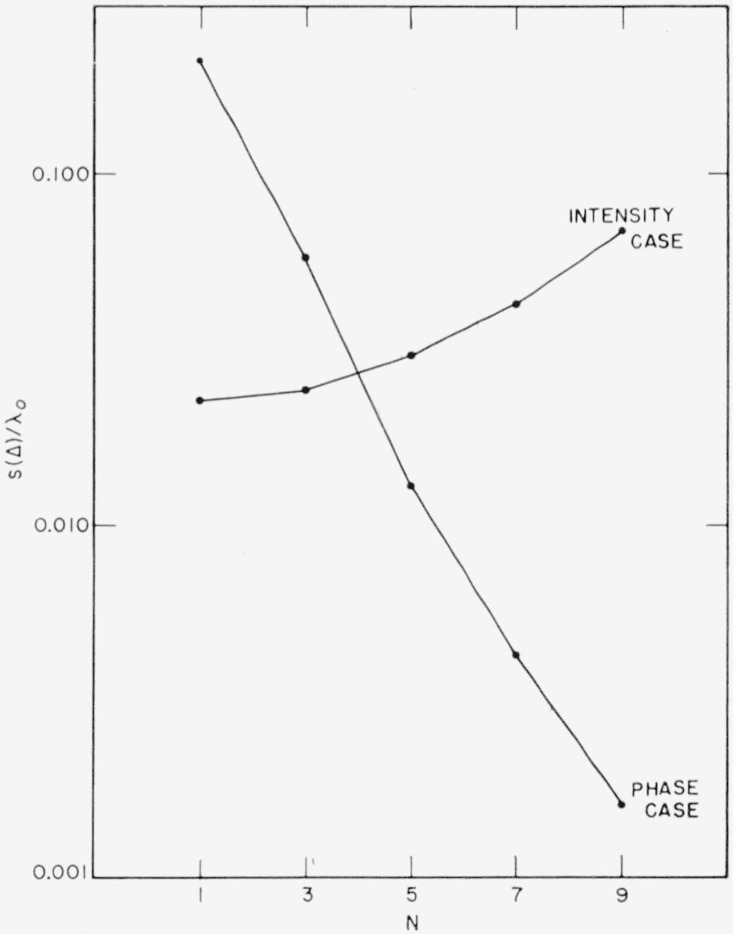
Thickness tolerances *s*(Δ) for mirrors or beam splitters to yield specified reflectance at λ_0_ within 1 percent (intensity case) and for interference filters to yield maximum transmission plus or minus one half their halfwidth (phase case) *ZnS*-*MgF*_2_-films.

Assume *f* = 0.99, corresponding to the rather strict requirement that theoretical reflectance has to be reproduced within 1 percent. The 0.99-widths of zinc sulphide-magnesium fluoride multilayers, taken from reflection curves as in figures 1a, b, c, are given in figure 7. Figure 8 shows the corresponding *s*(Δ)′s computed from (59) with the *A*(*β*) values of figure 5.

The obvious conclusion from figure 8 is that, even if very narrow tolerances are to be met, the production of a multilayer mirrow or beam splitter hardly presents any experimental difficulties. The permissible thickness error rises sharply as *N* increases so that, the more complicated the multilayer gets, the easier it becomes to produce its individual layers. In the example chosen, the permissible thickness error varies from 0.023 *λ*_0_ for the single film to 0.069 *λ*_0_ for the nine-layer stack, corresponding to as much as about 10, or 28 percent, respectively, of the nominal thickness of *λ*_0_/4. Simple monitoring systems should, therefore, be sufficient for obtaining experimentally the theoretical reflectances of which alternating multilayer coatings are capable.

### 5.3. Phase Case: Interference Filters

The energy transmittance of a Fabry-Perot interferometer is given by the familiar Airy formula [5],

$$T=\frac{\tau^{2}/{\left(1-\rho \right)}^{2}}{1+4\rho {\;\sin\;}^{2}\gamma /{\left(1-\rho \right)}^{2}},$$ (60)

with

γ=(π/λ)OPD. (61)

*ρ* and *τ* denote energy reflectance and transmittance of either interferometer plate, and *OPD* is the optical path difference between two successive beams, which at normal incidence is

OPD=2n(t+Δt),

where *n* is the refractive index of the spacing medium, *t* its geometrical thickness, and Δ*t* the change in path due to phase change upon reflection from one of the interferometer plates. By convention [6], the calculated value for the phase change represents an increase in optical path of

nt=(λ/2π)(μ2π−Φ),

*μ* being an integer. Thus,

γ=μ2π+2πnt/λ−Φ.

Consider an all-dielectric (nonabsorbing) first-order interference filter. Then

$$\begin{array}{l} \;\rho =1-\tau ,\\ nt=\lambda_{0}/2, \end{array}$$

and because of (11),

γ=μ2π+2β−Φ.

Therefore,

$$T={\left[1+\frac{4\rho }{{\left(1-\rho \right)}^{2}}{\;\sin\;}^{2}\left(2\beta -\Phi \right)\right]}^{-1}.$$ (62)

For the ideal interference filter, having ideal quarter wave multilayer coatings on either side of the spacer layer, one has Φ = 180° and therefore maximum transmission, *T* = 1, at *β* = 90° where

2β−Φ=0. (63)

The half width of the pass band,

$$2\Delta \beta_{HW}=2|90{}^{\circ}-\beta_{HW}|,$$ (64)

follows from *T* = ½, or

$$\;\sin\;(2\beta_{HW}-\Phi )=(1-\rho )/2\sqrt{\rho }.$$ (65)

Using the values of Φ and *ρ* calculated previously in this paper (and thereby assuming the somewhat simplified case of an air spacer), the half widths shown in figure 7 were obtained for interference filters with *N* = 1, 3, 5, 7, and 9 alternating zinc sulphide-magnesium fluoride layers on each side of the spacer.

Incorrect layer thicknesses will cause a phase change of Φ + *δ*Φ, rather than of Φ. As a result, the center of the pass band will be shifted from *β* = 90° to *β* = 90°+∇*β*, the maximum being again *T* = 1. One may obtain ∇*β* from (63), or

2(90°+∇β)=Φ+δΦ.

According to sec. 4, *δ*Φ is independent of *β*. In the neighborhood of *β* = 90°, Φ is a linear function of *β*,

Φ=−mβ+p

(see figs, 1a, b, c), with

p=(m+2)90°

because of Φ = 180° for *β* = 90°. Therefore,

2(90°+∇β)=−m(90°+∇β)+(m+2)90°+δΦ,

or

∇β=δΦ/(m+2). (66)

Allow a tolerance of one half the width of the pass band,

$$\nabla \beta =\pm \Delta \beta_{HW},$$ (67)

which, according to (66), corresponds to a tolerance on the phase shift upon reflection of

$$\delta \Phi =\pm (m+2)\Delta \beta_{HW}.$$ (68)

Transmittance at *λ*_0_, then, may depart from the desired value *T* = 1 by 50 percent.

With *δ*Φ from eq (68) substituted for *s* (*δ*Φ), eq (55) then provides the thickness tolerance

$$s(\Delta )=\pm (m+2)\Delta \beta_{HW}/A(\Phi ).$$ (69)

Using the values of *A*(Φ) and Δ*β_HW_* from figures 5 and 7, and with *m*’s taken from Φ–versus–*β*–curves as in figures 1a, b, c, the *s*(Δ)’s of figure 8 were obtained.

It is obvious from figure 8 that, in the phase case, monitoring film thicknesses is by far more demanding than in the intensity case. The permissible thickness errors decrease very rapidly as *N* increases so that production of the filter becomes increasingly difficult with increasing filter performance. For the widely used seven layer coatings on each side of the spacer, a tolerance of as little as *s*(Δ) = ±0.0043 *λ*_0_, or 1.7 percent of the nominal thickness of *λ*_0_/4, is required in the example chosen. For 9-layer coatings, the tolerance is even further reduced to *s*(Δ) = ±0.0016 *λ*_0_, or 0.65 percent of *λ*_0_/4. Compared hereto, the permissible *s*(Δ)’s for 7- and 9- layer reflectors are about 10 and 42 times greater in the example chosen for the intensity case; see figure 8.

The production of all-dielectric interference filters, therefore, requires monitoring equipment much more efficient than that sufficient for producing dielectric mirrors and beam splitters.

## 6. Comparison of Monitoring Techniques

A simple and widely used method of controlling layer thicknesses, first described by Dufour [7], is measuring with a photo cell and a galvanometer the intensity of a fairly monochromatic light beam reflected from the growing dielectric film, and ceasing evaporation whenever a maximum or minimum galvanometer deflection is reached. In this author’s experience, an accuracy of about ±6 percent of the desired thickness of a quarter wavelength of visible light can be obtained with this “single photo cell” technique, using as light source an incandescent lamp plus a gelatine filter of about 300 A half width. Somewhat better accuracies may be obtained by employing, instead of the simple gelatine filter, a narrow pass band interference filter or a monochromator. According to the results of sec. 5.2, therefore, this technique of controlling film thicknesses should be fully sufficient for the production of multilayer mirrors and beam splitters.

Provision has to be made, however, to fulfill eq (54), according to which each layer in the stack can be prepared with equal facility, and upon which the conclusions of sec. 5 were based. Towards the completion of a high reflection multilayer, the difference in reflectance caused by each additional layer is rapidly decreasing; see table 3. Direct monitoring of more than five or seven layers is, therefore, impossible with the described method. To overcome this difficulty, one may either use the technique of monitoring on separate glass plates only a few layers at a time [7], or employ a differential photometer such as described by Linberg and Irland [8].

If eq (54) is not fulfilled, appropriate weight factors *w_v_* must be applied so that

$$s(\Delta_{v})=w_{v}\;s(\Delta ),$$

instead of (54), and with *s* (Δ) being a suitable starting value. Equation (56) would then be changed to

$$A^{2}=\sum_{v=1}^{N}w_{v}^{2}a_{v}^{2},$$

and the results that follow would have to be altered accordingly. In view of the vast range of possible weight factors, however, their consideration is beyond the scope of this paper.

The accuracy of the single photo cell method is limited by the fact that it measures the change in reflectance with thickness and that, at the desired quarter wave thickness, this change is zero [7]. The method, therefore, is not likely to provide the high accurancies required for the production of dielectric interference filters.

Giaccmo and Jacquinot [9] have developed a more precise monitoring technique in which, rather than reflectance, its differential quotient with respect to wavelength is observed. At a quarter wave layer thickness, this differential quotient goes through zero, its change with thickness being a maximum. A similar but in practice simpler method was described by Traub [10]. The accuracy of these methods is better than 1 percent of the layer thickness [10]. According to sec. 5.3, the production of dielectric interference filters to within plus or minus one-half the width of their pass bands, therefore, appears to be possible with Giacomo and Jacquinot’s or Traub’s techniques. Equation (52) may be satisfied by using separate monitor glasses.

## Figures and Tables

**Table 1:** Effect of 10 percent thickness errors Δ on reflectance, at central wavelength λ_0_, of alternating quarter wave layers of ZnS and MgF_2_ between air and a glass substrate (*n_0_* = 1, *n_H_* = 2.3, *n_L_* = 1.38, *n_s_* = 1.52); ZnS bottom layer

Type of Coating	Reflectance at *λ*_0_	
	Δ=0	Δ = λ_0_/40

Single ZnS film…………	0.306	0.302
Five-layer, error in top layer…………	.861	.859
Nine-layer, error in central layer…………	.981	.980

**Table 2:** Individual and total errors

Δ*_v_* = thickness error of *v*th layer in percent of *λ*_0_/4; *δβ_v_*, *δϕ_v_* = results of Δ*_v_* if occur ring alone; *δβ*, *δϕ* = results of all Δ*_v_*′s occurring simultaneously. ZnS-MgF2-films.				
Type of Coating	Σ*δβ_v_*	*δβ*	Σ*δϕ_v_*	*δϕ*

Five-layer, Δ_1_ = 10%, Δ_2_=5%, Δ_4_ = −5%, Δ_5_ = −10%……	−0.7°	−0.6°	−8.8°	−8.7°
Seven-layer, Δ_1_ = Δ_7_ = 5%, Δ_4_= 10%……………	−1.7°	−2.0°	−5.6°	−5.6°
Nine-layer, all Δ*_v_*′s=10%…	−8.0°	−8.2°	−19.2°	−19.0°

**Table. 3:** Reflectance ρ of N alternating layers of ZnS and MgF_2_ between air and a glass substrate.

ZnS bottom layer.					
N	*ρ*	Change in *ρ* caused by Nth layer	N	*ρ*	Change in *ρ* caused by Nth layer

	%	%		%	%
0	4.3		6	75.3	−10.8
1	30.6	+26.3	7	94.8	+19.5
2	8.6	−22.0	8	90.3	−4.5
3	66.0	+57.4	9	98.1	+7.8
4	45.0	−21.0	10	96.9	−1.2
5	86.1	+41.1	11	99.3	+2.4

## Footnotes

1 Figures in brackets indicate the literature references at the end of this paper.

2 Table 1 was obtained by exact computation. Because of the smallness of the effect, the approximation is not accurate enough here.

## References

[1] Heavens OS (1954). J Opt Soc Am.

[2] Giacomo P (1956). Rev optique.

[3] Koehler WF (1955). J Opt Soc Am.

[4] Mielenz KD (1959). J Research NBS.

[5] Steudel A (1957). Naturwissenschaften.

[6] Koester CJ (1960). J Research NBS.

[7] Dufour C (1948). Le Vide.

[8] Linberg VL, Irland MJ (1955). J Opt Soc Am.

[9] Giacomo P, Jacquinot P (1952). J phys radium.

[10] Traub AC (1956). J Opt Soc Am.

